# From the Streets to the Judicial Evidence: Determination of Traditional Illicit Substances in Drug Seizures by a Rapid and Sensitive UHPLC-MS/MS-Based Platform

**DOI:** 10.3390/molecules28010164

**Published:** 2022-12-25

**Authors:** Fabio Gosetti, Viviana Consonni, Davide Ballabio, Marco Emilio Orlandi, Angelo Amodio, Maria Valeria Picci, Marco Visentin, Veronica Termopoli

**Affiliations:** 1 Department of Earth and Environmental Sciences, University of Milano-Bicocca, Piazza della Scienza 1, 20126 Milan, Italy; 2 Legione Carabinieri Lombardia, Comando Provinciale di Milano Sezione, Investigazioni Scientifiche L.A.S.S., Via V. Monti 58, 20145 Milan, Italy; 3 Gabinetto Regionale di Polizia Scientifica per la Lombardia, Polizia di Stato, Via Fatebenefratelli 11, 20121 Milan, Italy

**Keywords:** street seizures, UHPLC-MS/MS, illicit substances, psychotropic substances, drugs of abuse mass spectrometry

## Abstract

According to the 2021 World Drug Report, around 275 million people use drugs of abuse, and 36 million people suffer from addiction, fostering a thriving market for illicit substances. In Italy, 30,083 people were reported to the Judicial Authority for offenses in violation of the Italian Law D.P.R. 309/1990. These offences are sentenced after a qualitative–quantitative analysis of seized materials. Given the large quantity of seized drugs and the need to perform accurate analytical determinations, Italian forensic laboratories struggle to complete analyses in a short time, delaying the entire reporting process needed to achieve sentencing. For this purpose, an UHPLC-MS/MS-based platform was developed at the University of Milano-Bicocca to support law-enforcement authorities. Software was designed to easily manage street seizure acquisition, documentation registration, and sampling. A sensitive UHPLC-MS/MS method was fully validated for the quantification of the traditional illicit substances (cocaine, heroin, 6-MAM, morphine, amphetamine, methamphetamine, MDMA, ketamine, GHB, GBL, LSD, trans-∆9-THC, and THCA) at the ppb level. The final report is relayed to the Prefecture in 3–4 days, even within 24 h for urgent requests. The platform allows for semi-automatic data handling to minimize erroneous results for an accurate report generation by standardized procedures.

## 1. Introduction

Consumption and trafficking of illicit substances are among the most significant worldwide concerns [1]. According to the 2021 World Drug Report, around 275 million people continue using drugs due to the psychotropic, narcotic, or excitant properties of these natural or synthetic pharmacologically active substances. After consumption, the perception of feeling stronger, more disinhibited, or satisfied leads consumers to increase the use of these substances, resulting in over 36 million people suffering from drug use disorders and addiction. The increasing demand caused a thriving market for illegal substances, where criminals and criminal organizations manage the drug trade for their lucrative scope at the expense of addicted consumers [2]. In the early 2020s, the global pandemic situation slowed transnational drug trafficking because of the health crisis on trade transitions. However, during the second half of 2020, the criminal network adapted logistics to travel restrictions and border closures, returning the market to pre-pandemic levels [3,4]. The 2021 National Drug Report of the Italian Department of Drug Policy indicated that 30,083 people were reported to the Judicial Authority for criminal offenses committed in violation of Italian Law D.P.R. 309/1990 (9 October 1990 and subsequently amended in 2006, L49/06) [5,6,7]. This law regulates prosecution or sentencing for the possession, trafficking, and production of substances included in dedicated Ministerial tables comprising controlled or banned molecules and their precursors. These tables are constantly updated by the Italian Ministry of Health following the European Monitoring Centre for Drugs and Drug Addiction (EMCDDA) indications [8,9]. The above-mentioned violations include 45% of the drug-related offenses for the possession of cocaine/crack, 41% cannabis and derivatives, 8% heroin/other opiates, and 1.3% synthetic substances. Focusing on Lombardy, one of the Italian areas with a prosperous and prominent drug trafficking market, the law-enforcement authorities seized 10,713 kg of illicit substances, including cocaine, heroin, hashish, marijuana, and synthetic drugs.

To classify the severity of the sanctions for drug-related crimes, the Judicial Authority requires qualitative–quantitative analyses of seized materials, considering the quality and quantity of the seizures. They are needed to determine the amount of active ingredient in seized materials, expressed as Threshold Amounts (TA) or Maximum Permitted amount (MPA), and defined according to the maximum number of single doses of illicit substances (in terms of mg) allowed for daily personal use [10,11]. The offenders caught with a number of scheduled substances higher than the maximum permitted doses for personal use are subjected to administrative or penal sanctions for possessing or selling illicit substances. For this purpose, after the first qualitative on-site drug-checking procedure achieved with colorimetric tests by law-enforcement officers, the accurate analytical identification and quantification of active ingredients in seizures are performed in dedicated analytical laboratories [12,13,14].

Gas chromatography (GC) coupled to flame ionization detector (FID) or mass spectrometry (MS) is the most used technique in this framework due to its extreme reliability and simplicity [14,15,16,17,18]. In recent years, liquid chromatography–tandem mass spectrometry (LC-MS/MS) has represented a powerful tool for the simultaneous detection of several illicit substances, mainly because of the possibility of coupling with dilute and shoot sample preparation [19,20,21,22,23,24]. Following the principle of “less sample handling”, this allows for the minimization of sample manipulation and the speeding up of the entire sample preparation procedure. However, given the large quantity of seized drugs and the need to perform accurate analytical determinations in a short time, the scientific laboratories of the “Gabinetto Regionale di Polizia Scientifica per la Lombardia” and “Comando Provinciale dell’ Arma dei Carabinieri di Milano” are subjected to an overwhelming workload. This affects the analysis timeline, delaying the entire reporting process needed to achieve sentencing (an average time of 20 days per person reported under arrest and 150 days for people registered on the loose). 

To support the law-enforcement authorities and reduce the response time of analyses, since September 2021 the Novel Psychoactive Substances (NPS) Laboratory of the University of Milano-Bicocca has been involved in a collaboration with the Prefecture of Milano to determine the quality and the quantity of the most abused illicit substances in street seizures. For this purpose, dedicated custom-made software was developed to manage the judicial documentation, ensuring the traceability and transparency of the entire supply chain required by the current Italian regulations. It allows the NPS Lab workers to acquire all needed information at the sampling registration step, such as the origin of the seized material, penalty process, gross and net weight, morphological characterization, packaging description and supposed substance. After seizure registration and sampling, a fast sample preparation procedure followed by a sensitive UHPLC-MS/MS method was developed to quantify 13 out of the most abused illicit substances in street seizures. This method was fully validated at the University of Milano-Bicocca and allows the simultaneous extraction and quantification of cocaine, heroin, 6-MAM, morphine, amphetamine, methamphetamine, MDMA, ketamine, GHB, GBL, LSD, trans-∆9-THC, and THCA in solid and liquid samples in a very short time of analysis (10 min for the sample preparation and 8 min for the UHPLC-MS/MS analysis, including column re-equilibration).

## 2. Results and Discussion

### 2.1. Development of the UHPLC-MS/MS Method

The development of a suitable analytical method for the determination of the investigated psychotropic substances considered the simultaneous presence of both acidic (e.g., THCA) and basic (e.g., cocaine, MDMA, etc.) substances, highly polar substances such as GBL and/or GHB sodium salt (whose direct determination would have been impossible by GC-MS), and lipophilic substances such as THC. It follows that the final chromatographic and mass spectrometric conditions obtained were the result of the best trade-off among method sensitivity, chromatographic resolution, and matrix effect.

Both methanol and acetonitrile (ACN) were tested in a mixture with water; at the same elution gradient, it was observed that ACN reduced the intensities of all chromatographic peaks by about 1/10 compared with the use of methanol, which, however, reduced the S/N of the qualifier transition of GBL.

The mobile phase was tested by adding either formic acid or acetic acid in concentrations up to 0.1%. Acetic acid was preferred because of the most satisfactory S/N of each chromatographic peak. The highest intensities of all analyte peaks, without compromising the decreased signal of the acidic species THCA ionized in NI mode, were achieved with an addition of 0.02% of acetic acid. The addition of even small concentrations (1–2 mM) of either ammonium formate or ammonium acetate enhanced the signal intensity of most analytes but inhibited the ionization of both GHB and GBL; therefore, they could not be used in the mobile phase.

The chromatographic separation was optimized by thoroughly adjusting the UHPLC parameters such as flow rate, symmetric peaks, and autosampler and column temperatures. To avoid possible degradation of the more labile analytes (e.g., trans-∆9-THC and THCA) during the analysis batch, the autosampler temperature was always kept at 4 °C.

Optimization of the MS/MS method was performed for all the target compounds using the standard solution mixture at a concentration of 100 ng/mL. The precursor ion of every compound was selected as the most abundant peak of each spectrum. Different collision energies (from 5 to 50 V) were tested to perform the fragmentation. For each compound, the collision energies that yielded the one precursor–three products ion transitions with the best signal intensity were chosen (except for GBL and GHB-D6 for which only two transitions were selected) to build the SRM acquisition method, addressing the requirements of the 2021/808/EC [25]. The most intense transition was chosen as the quantifier, and the second and third most intense ones were chosen as the qualifiers. The SRM transitions, the optimized compound-dependent parameters such as Q1 and Q3 voltage potentials, collision energies (CE), and the related deuterated IS are reported in Table S1. All the analytes were ionized in PI mode, with the only exception being THCA, which ionized in the NI mode. The SRM transitions of each analyte were monitored only in defined time windows of 60 s centered on the related retention time. The total cycle time was 0.424 s, including a polarity switching of 5 ms and 23 events of 0.018 s. 

**Figure 1:**
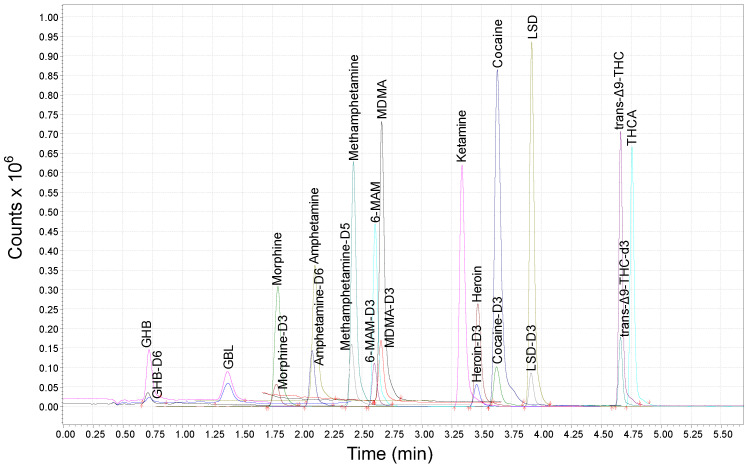
Chromatographic separation of a standard mixture of the target compounds (each at 50.0 ng mL^−1^) and their relative ISs in SRM acquisition mode.

A chromatographic separation of the target compounds and their ISs is illustrated in Figure 1. It is important to note the excellent profile of the peaks of all the analytes in a very short elution time (lower than 5 min).

### 2.2. Method Validation

A fit-for-purpose method validation was performed based on the standard practices for method validation in drug analysis. The method was validated considering LODs, LOQs, linearity range, accuracy, repeatability, reproducibility, recovery, and matrix effects.

For all analyzed samples, the active ingredient quantification was based on an eight-point calibration curve (LOQ, 0.1, 0.5, 1.0, 10.0, 50.0, 100, 250 ng/mL) using mixture standard solutions freshly prepared every week and added with their corresponding ISs (Table S1) at a concentration of 10 ng/mL. Internal calibration models with weighting factor 1/x were calculated, using the peak area ratio of the “quantifier” transition signal and the corresponding IS as the dependent variable y and the ratio of the standard over the IS concentration as the independent variable x. The quality of linear regression models was high, with R^2^ always greater than 0.9945 (Table 1). Accuracy at the eight concentration levels was always within the acceptable range of 85.6–108.7%.

The limits of detection (LOD) and quantitation (LOQ) were calculated according to the International Council for Harmonization of Technical Requirements for Pharmaceuticals for Human Use (ICH) [26] as the analyte concentration giving a signal of 3.3 s_B_/b and 10 s_B_/b, respectively, s_B_ being the blank standard deviation equal to the residual standard deviation (s_y_/x), and b the calibration model slope. The obtained LODs and LOQs were at low-ppb levels, as shown in Table 1, and their values were more than satisfactory, considering the high concentrations of active ingredients in seized drug samples.

The obtained LODs and LOQs were lower than those reported in the literature for similar applications with respect to the investigated compounds (Table 2). In addition, the analysis time was among the lowest of those reported in Table 2. 

Carry-over and memory effects were carefully evaluated. For this purpose, blank sample injections of the mobile phase after the injection of the standard solution mixture at the highest concentration level of the calibration plot were acquired. The presence of the peak of cocaine at the concentration close to the respective LOQ value was observed. Carry-over was overcome by using a proper washing solution for the autosampler, which consisted of a mixture of MeOH/isopropanol/water (80/10/10 (*v*/*v*/*v*)) used to clean both the needle and the pump port before and after each injection for 5 s. In addition, no memory effect was found thanks to the above-mentioned autosampler washing step and the washing of the chromatographic column with 100% of organic solvent (B) for 2 min at an increased flow-rate of 0.65 mL/min. This allowed to the chromatographic column to be fully cleaned of any impurities or residues that may have been present in the injected samples. Selectivity was investigated by the analysis of 10 consecutive injections of mobile phase (blank) samples. No interfering species were observed at the retention time of the analytes or IS in the analyzed blank samples.

In order to use the method in routine analyses, a specific control step must be applied to verify the method stability over time [26]. For this purpose, a quality control (QC) solution at 25.0 ng/mL was analyzed every 20 injections of each sample batch as part of the quality control process. All the results obtained for the QC solutions lay within the ±σ control limits of the calibration plots. Intra-day (i.e., repeatability) and inter-day (i.e., intermediate) precisions on concentration and retention time were determined by analyzing 5 replicated QC samples per day (*n* = 5) and for 7 days (*n* = 35), respectively. The obtained intra- and inter-day precisions in terms of relative standard deviation (RSD%) are reported in Table 1. They were lower than 4.1% and 12.6%, respectively, for the concentration and lower than 0.6% and 1.9%, respectively, for the retention time.

The evaluation of matrix effects is a crucial step in the analysis of challenging samples, especially if the erroneous quantification of analytes can lead to a wrong decision in judicial processes [27,28,29,30]. The addition of isotopic standards compensates for the possible presence of matrix effects, and the quantification was performed by internal calibration. As the matrices of the analyzed samples had very diverse nature (e.g., powders, agglomerates, inflorescences, oils, tobacco, cannabis resins, liquids, etc.), the labeled standards of the various psychotropic substances were used to calculate the recovery by fortifying them within the different matrices subjected to the extraction process (paragraph 2). Therefore, samples of known composition were fortified with 50.0, 250.0, and 500.0 µL of the respective labeled standard at 1000 mg/L to determine recovery directly on the matrix by the external calibration model, which was calculated in the same range of the internal calibration model. Each extraction was repeated three times.

Recovery values obtained for each analyte spanned from 94.3 ± 0.5% to 109.1 ± 0.7%, demonstrating the suitability of the developed extraction procedure even in the presence of complex matrices such as marijuana and hashish. Both types of samples gave the same recovery value as THC and THCA. The recovery values were reproducible and independent of the analyte concentration within the explored concentration range. According to the t-test (95% confidence level), there was no significant difference among the three obtained recovery values; therefore, the average recovery is reported in Table 1.

## 3. Analytical Results of Street Seizures

**Figure 2:**
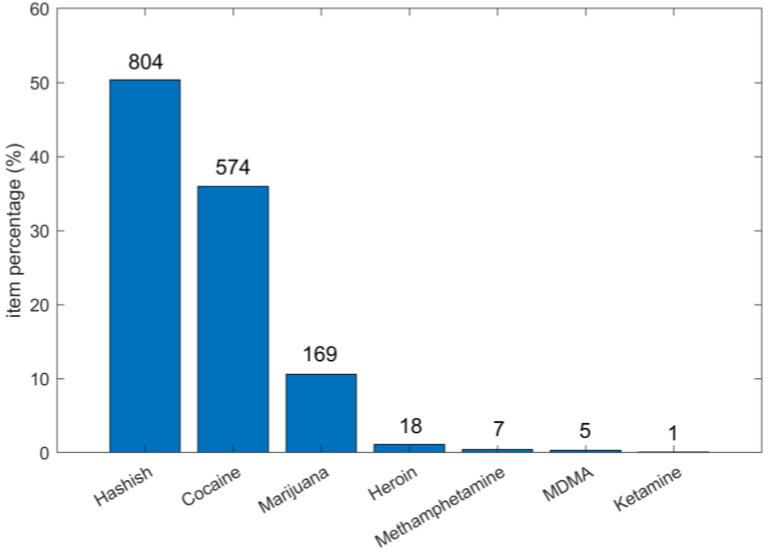
Pareto plot of item percentage for the different categories of street seizures. The total number of items per category is shown at the top of each bar.

From September 2021 to June 2022, a total of 311 seizures was registered and analyzed at the NPS Laboratory by the above-mentioned UHPLC-MS/MS method. The processed seizures included overall 1587 items, which were classified as follows: 1324 items as homogeneous, and 263 items as heterogeneous. From the item distribution in the different categories of drugs of abuse (Figure 2), it was noticed that most of the registrations were related to hashish (804) and cocaine (574) seizures; marijuana (169) was less frequent, and only a few seizures of heroin (18), methamphetamine (7), MDMA (5), and ketamine (1) were received. For nine items, none of the targeted psychoactive substances was detected. 

Most of the seizures were of small to medium size; in fact, around half of the registered items (53.5%) had a net weight less than 1 g; 29.4% were between 1 and 10 g, 15.7% were between 10 and 100 g, and just 1.4% were more than 100 g. 

**Figure 3:**
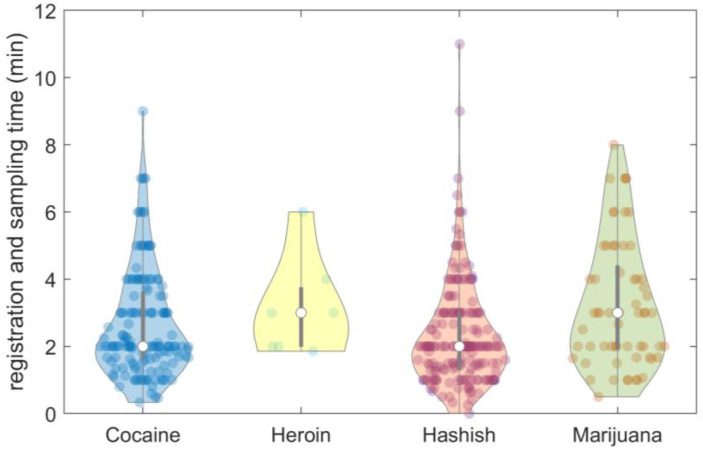
Violin plot of registration and sampling time for the most frequent types of street seizures.

Figure 3 shows the distribution of the time required for registering and sampling the most frequent types of street seizures [31]. On average, the time was around 2 min for cocaine and hashish, while marijuana and heroin required an average time of around 3 min. Time variability is due to the number and features of the items included in each street seizure. However, the entire process of registration and sampling required less than 5 min for the majority of seizures.

**Figure 4:**
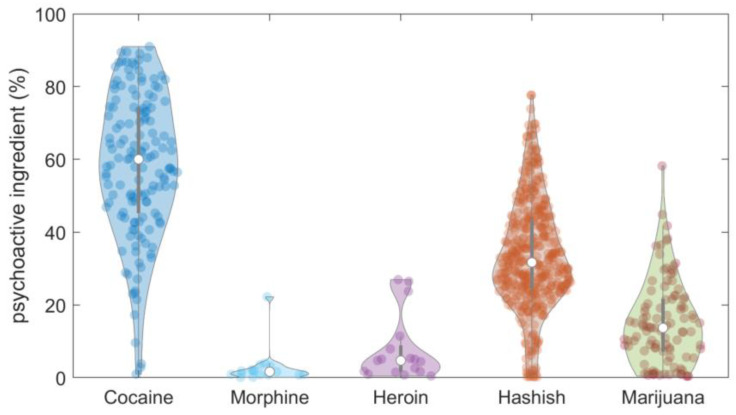
Violin plot of psychoactive ingredient (%) for the most frequent types of street seizures.

Looking at the distribution of the psychoactive ingredient (%) for the most frequent types of street seizures (Figure 4), i cocaine on average had a high percentage of psychoactive ingredient (around 60%), as expected. The very large dispersion of the ingredient percentage observed in the set of analyzed cocaine samples may have been related to the very diverse nature of the received seizures. The average percentage of trans-∆9-THC in hashish (34%) was larger than that in marijuana (15%). Finally, heroin and morphine had the lowest percentages of psychoactive ingredient (less than 10%).

## 4. Materials and Methods

### 4.1. From Seizures Registration to Final Report Generation

**Figure 5:**
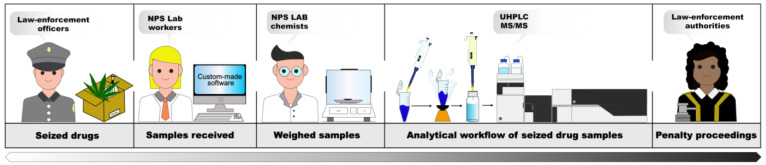
Workflow of the seizure analysis process.

The workflow of the entire process is shown in Figure 5. The first stage concerns seizure registration by custom-made software designed to collect all the meaningful information, followed by the weighing, sampling, and analysis steps. All the registration and analysis data are processed to generate the final report in a semi-automatic process by standardized procedures. 

### 4.2. Street Seizure Registration and Sample Collection

**Figure 6:**
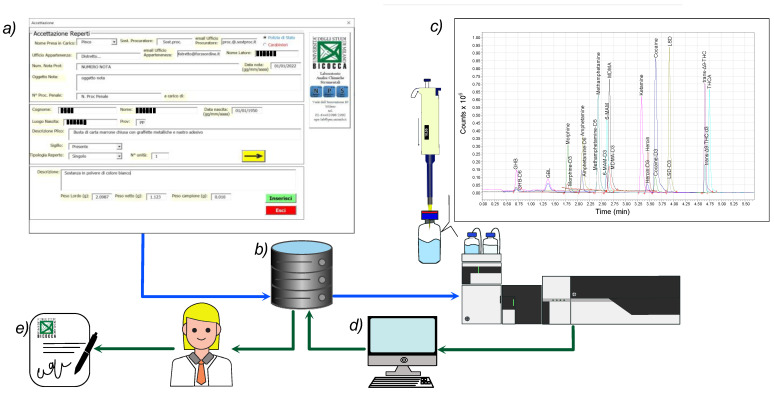
Schematic representation of the semi-automatic process for report generation, including data handling, analytical measurements, and results acquisition. (**a**) Screenshot of the software for seizure registration; (**b**) NPS Lab database with a two-way connection between registration software and UHPLC-MS/MS instrumentation software; (**c**) sample extraction and UHPLC-MS/MS determination; (**d**) data elaboration and final reporting; (**e**) report relayed to the law-enforcement authorities.

All street seizures were provided by the law-enforcement authorities operating in the municipal area of Milano (Italy) from September 2021 to June 2022. Registration of the seizures comprising the chain of custody was performed according to the Italian regulations under the following conditions:

- Registration. A dedicated custom-made software developed at the NPS Laboratory (Figure 6a) allows for easy registration of all the meaningful information about seized materials. To every single seizure, a unique alphanumeric ID is assigned, and a detailed descriptive technical sheet is generated. After seizure coding, the NPS workers register visual and morphological descriptions of the seizure-relevant aspects, e.g., type and shape of wrapping materials, color, odor, forms, marks, total number of similar items of each type of supposed substance, their gross and net weights, and any other characteristic that can be considered essential. The gross and net weights of each single item are measured and registered up to a maximum of 20 similar items. For more than 20 similar items, the total gross and net weights of the seizure are estimated based on the average weight of the sampled items and the total number of items in the seizure. One or more digital images of the seizures are taken before and after unpacking. The photographic appendix is a valuable part of the final report, especially for the prosecutor’s data handling and decision. Any other relevant visual aspect considered to be of interest to the prosecutor’s findings that could emerge during the laboratory processing of the seizure is recorded as well.

Sample collection. Generally, qualitative, and quantitative methods used in forensic drug analysis laboratories require very small aliquots of material, implying that these small aliquots must be representative of the original bulk material [32,33]. Sample collection is performed in accordance with the guidelines for the sampling of illicit drugs for quantitative analysis of the European Network of Forensic Science Institutes (ENFSI) [34], and the specific guidelines provided by the G.P.R.S Lombardia and L.A.S.S.—Nucleo Investigativo Comando Provinciale dell’ Arma dei Carabinieri. When multiple items in the same seizure are present, they are divided into subgroups based on the nature of the material and the morphological and packaging similarities (e.g., size, shape, color, supposed substance, wrapping, marks, etc.). In particular, two subgroups of seizures are defined: homogeneous and heterogeneous. For the latter, each item is weighted and sampled, whereas for homogeneous seizures, the following rules based on incremental sampling apply:

From 1 to 10 similar items, only 1 item is randomly chosen, weighed and sampled;

From 11 to 20 similar items, 3 items are randomly chosen, weighed and sampled;

From 21 to 100 similar items, 5 items are randomly chosen, weighed, and sampled;

For more than 100 similar items, 10 items are randomly chosen, weighed, and sampled.

Before sampling, the representative items are carefully homogenized in a mortar to make the sample collection as most representative as possible. For cannabis resin (i.e., hashish), representative items are gently cut to collect the sample at the internal midpoint of the material. This sampling point is selected after a preliminary analysis of the concentrations of the psychoactive ingredient, which are determined in five different spots of the seizure items: 1 on the surface center, 2 on the surface edges, 1 in the inner central point, and 1 in the middle from the surface to the center. It has been observed that the ingredient concentration increases from the surface to the internal center; therefore, sampling is always performed at the internal midpoint of the item

The weighted samples are finally stored in a closed conical tube until analysis. The remaining seizure material is resealed in the original wrapper and returned in sealed stamped envelopes along with a signed delivery report. Information is also collected and saved in the NPS Lab database for the subsequent analysis steps.

### 4.3. Chemical and Reagents

Certified standard materials of cocaine hydrochloride, heroin hydrochloride monohydrate, 6-acetylmorphine hydrochloride trihydrate (6-MAM), morphine hydrochloride, amphetamine hydrochloride, methamphetamine hydrochloride, d,l-3,4-methylenedioxymethamphetamine (MDMA) hydrochloride, ketamine hydrochloride, sodium gamma-hydroxybutyrate (GHB), gamma-butyrolactone (GBL), d-lysergic acid N,N-diethylamide (LSD), (–)-trans-∆9-tetrahydrocannabinol (THC), (–)-trans-∆9-THC carboxylic acid (THCA), and their deuterated analogues (used as internal standards, IS) (cocaine-D3 hydrochloride, heroin-D3 hydrochloride, 6-MAM-D3 hydrochloride, morphine-D3 hydrochloride, amphetamine-D6 hydrochloride, methamphetamine-D5 hydrochloride, MDMA-D3 hydrochloride, GHB-D6 sodium salt, LSD-D3, THC-D3) were purchased from Chebios (Rome, Italy). The complete list of the target compounds used for method validation and their relative ISs are reported in Table S1. Ultrapure water, methanol, and acetic acid (LC-MS purity grade) were purchased from Carlo Erba (Milan, Italy). Conical PE tubes used for storing seizure samples were obtained from Carlo Erba (Milan, Italy). Individual standard stock solutions were prepared by dissolving each standard in methanol with a concentration of 100.0 µg/mL and stored at −20 °C until use. Working mixture standard solutions containing all selected compounds and their deuterated analogues were freshly prepared by appropriate dilution of the stock standard solution with 98/2 (*v*/*v*) water/methanol acidified with 0.02% acetic acid for the quality control sample (QC). 

### 4.4. Sample Preparation Procedure

A volume of 5.00 mL of methanol was added to accurately weighed 10.0 mg of sample, vortexed for 30 s, and sonicated for 5 min. Then, 5.0 µL of each extracted sample was further diluted to the final volume of 5.00 mL by adding methanol and 50.0 µL of the deuterated ISs mixture in methanol at a concentration of 10.0 µg/mL. The solution was vortexed again for 30 s and filtered on a PTFE filter (0.22 µm). The sample was further diluted 1/10 (*v*/*v*) with a mixture of water/methanol 98/2 (*v*/*v*), both acidified with 0.02% acetic acid, and subjected to UHPLC-MS/MS analysis.

### 4.5. UHPLC-MS/MS Conditions

The UHPLC–MS/MS analyses were performed using a Nexera X2 Liquid Chromatography (Shimadzu, Kyoto, Japan) system, equipped with a DGU-20A3R degasser, two LC-30AD pumps, an SIL-30AC autosampler, a CTO-20AC column compartment, and a CMB-20 system. The system was interfaced with an LC-MS 8060 triple quadrupole mass spectrometer (Shimadzu, Kyoto, Japan) by an ESI ion source. The UHPLC-MS/MS data were acquired and processed with LabSolutions LCMS ver. 5.99 (Shimadzu Corporation, Kyoto, Japan) and LabSolutions Insight ver. 3.7 (Shimadzu Corporation, Kyoto, Japan) software. The stationary phase was a Raptor Biphenyl column (100 × 2.1 mm, 1.8 µm) acquired from Restek (Milan, Italy), and a Raptor Biphenyl UHPLC (5 × 2.1 mm) was used as a guard column. The mobile phase was a mixture of (A) water/methanol 98/2 (*v*/*v*) and (B) methanol/water 98/2 (*v*/*v*), both with the addition of 0.02% of acetic acid, eluting at a flow rate of 0.45 mL min^−1^ min under the following gradient conditions: 0.0–0.3 min 5% B, 0.3–2.2 min 43% B, 2.2–2.8 min 43% B, 4.0–6.0 min 100% B (increasing the flow rate up to 0.65 mL min^−1^), 6.1 min 5%. B (decreasing again the flow rate at 0.45 mLmin^−1^). The final run time was 8.0 min, including the re-equilibration step. The injection volume was 1.0 µL, and the oven and autosampler temperatures were set at 45 °C and 4 °C, respectively.

The ESI worked both in positive (PI) and negative polarity ion mode (NI) and the mass spectrometer was used in selected reaction monitoring (SRM) mode. The optimized instrumental parameters were set as follows: nebulizing gas flow 3.0 L/min; heating gas flow 10 Lmin^−1^; drying gas flow 10 Lmin^−1^; interface temperature 300 °C; DL temperature 250 °C; heat block temperature 400 °C. The unit mass resolution was established and maintained in each mass-resolving quadrupole by keeping a full width at half-maximum (FWHM) of approximately 0.7 u.

### 4.6. Final Report Generation

In the final stage of the seizure analysis workflow, UHPLC-MS/MS-acquired analytical results were integrated with the registration and sampling data, retrieved from the NPS Lab database, to calculate the percentage of illegal psychoactive ingredients contained in each seizure (Figure 6). The number of doses associated with the total net weight was calculated referring to the permitted limit values for each substance [8,9,10]. In this way, it was possible to quickly and easily associate with the analysis results the data collected during registration to obtain a complete and detailed final report of all useful information for law enforcement authorities.

## 5. Conclusions

This study described the platform designed at the NPS Lab of the University of Milano-Bicocca to automate and standardize the entire acquisition process of street seizures, from registration and sampling to sample analysis and reporting. The custom-made software together with a fast and reliable UHPLC-MS/MS method allowed for the delivery of the analytical reports to the judicial authorities in 3–4 days, and the processing of urgent requests in 24 h. The developed and fully validated UHPLC–MS/MS-based platform was considered appropriate for quantifying the most abused illicit drugs and ensuring a robust and efficient procedure for accurately quantifying selected psychoactive substances, even in the presence of complex matrices. The custom-made software allows for easy and rapid access to the built database for rapid registration and report processes.

As a future goal, the method will be gradually implemented with the increasing number of new psychoactive substances (NPS) present on the market, drugs that are not controlled by international drug convention yet, but which may pose a serious threat to public health.

## Figures and Tables

**Table 1:** LOD, LOQ, R^2^, and intra- and inter-day RSD (%) of concentration and average recovery for each analyte.

Compound	LOD (ng mL^−1^)	LOQ (ng mL^−1^)	R^2^	RSD% Intra-Day (*n* = 5)	RSD% Inter-Day (*n* = 35)	Recovery (%)
6-MAM	0.01	0.04	0.9965	0.9	11.3	105.3 ± 0.7
Amphetamine	0.12	0.41	0.9998	0.1	10.4	101.1 ± 0.8
Cocaine	0.01	0.04	0.9989	4.1	10.7	106.1 ± 1.3
Heroin	0.01	0.02	0.9991	0.4	8.7	96.5 ± 0.9
GBL	1.02	3.02	0.9978	2.6	8.1	109.1 ± 0.7
GHB	0.11	0.32	0.9994	0.8	10.3	109.1 ± 0.7
Ketamine	0.01	0.05	0.9945	0.1	9.7	105.5 ± 0.8
LSD	0.01	0.03	0.9992	2.7	8.1	94.5 ± 0.7
MDMA	0.04	0.12	0.9963	0.7	12.6	105.5 ± 0.8
Methamphetamine	0.04	0.13	0.9992	3.4	11.6	105.7 ± 0.3
Morphine	0.09	0.30	0.9991	1.3	2.3	96.1 ± 0.1
Trans-∆9-THC	0.04	0.47	0.9968	2.6	8.5	94.3 ± 0.5 ^a^ 94.3 ± 0.3 ^b^
THCA	0.09	0.31	0.9983	2.1	8.0	94.3 ± 0.5

^a^ marijuana. ^b^ hashish.

**Table 2:** Comparison with other methods reported in the literature.

Compound	Street Seizures	Extraction Solvent (Volume)	Instrumentation	Run Time (min)	LODs	LOQs	Ref.
Trans-∆9-THC	Herbal and resin cannabis	Cyclohexane (9 mL)	GC-FID	12	3 ng µL^−1^	4.2 ng µL^−1^	[11]
Heroin	Powder	ACN (Not declared)			4.5 ng µL^−1^	5.5 ng µL^−1^	
Cocaine	Powder	ACN (Not declared)			3.5 ng µL^−1^	4.5 ng µL^−1^	
MDMA (and other 6 compounds)	Tablets	Absolute ethanol (20 mL)	GC-MS	33	32 ng µL^−1^	Not declared	[14]
Cocaine	Powder	Methanol/ Chloroform (Not declared)	GC-FID	13	1.8 ng µL^−1^	5.6 ng µL^−1^	[16]
Cocaine	Powder	Methanol (10 mL)	Portable GC-MS	15	From 40 ng µL^−1^ to 100 ng µL^−1^	Not declared	[17]
Heroin							
Methamphetamine (and other 21 compounds)							
Cocaine	Solid and liquid materials	ACN (5 mL)	LC-MS/MS	15	1.67 ng mL^−1^	5 ng mL^−1^	[19]
MDMA							
Trans-∆9-THC							
Heroin							
Ketamine							
6-MAM (and other 31 compounds)							
Heroin (and other 3 compounds)	Powder	Not declared	LC-MS/MS	6	0.4 mg L^−1^	1 mg L^−1^	[21]
Cocaine	Solid and liquid materials	Methanol (10 mL)	LC-MS/MS	8	From 0.01 ng mL^−1^ to 1.02 ng mL^−1^	From 0.04 ng/mL to 3.04 ng/mL	This validated method
Heroin							
6-MAM							
Amphetamine							
GHB							
GBL							
Ketamine							
LSD							
MDMA							
Methamphetamine							
Morphine							
Trans-∆9-THC							
THCA							

## Data Availability

Not applicable.

## Supplementary Materials

The following supporting information can be downloaded at https://www.mdpi.com/article/10.3390/molecules28010164/s1. Table S1: SRM transitions (Q1 and Q3 *m*/*z*), ionization mode (PI for positive ion and NI for negative ion), retention time, and mass spectrometry parameters.
