# Supplementary material for: From the Streets to the Judicial Evidence: Determination of Traditional Illicit Substances in Drug Seizures by a Rapid and Sensitive UHPLC-MS/MS-Based Platform
Source: Molecules. 2022 Dec 25;28(1):164. doi: 10.3390/molecules28010164 (PMC9822244; doi:10.3390/molecules28010164)
Supplement: Supplementary file 1 [file molecules-28-00164-s001.zip › molecules-2109389-supplementary.pdf]

Table S1. SRM transitions (Q1 and Q3 m/z), ionization mode (PI for positive ion and NI for negative ion), retention time and mass spectrometry parameters: Q1 Pre Bias, CE, Q3 Pre Bias. The internal standard (IS) coupled with each analyte is reported in the last column.

Compound	Ionization mode	RT (min)	Q1 (*m/z*)	Q3 (*m/z*)	Q1 Pre Bias (V)	CE (V)	Q3 Pre Bias (V)	IS
6-MAM	PI	2.60	328	165/211/193	-17/-23/-17	-45/-28/-30	-30 / -25 / -22	6-MAM-D3
6-MAM-D3	PI	2.60	331	165/211/193	-26/-18/-24	-42/-28/-29	-18/-23/-22	-
Amphetamine	PI	2.11	136	91/65/119	-28/-10/-24	-23/-37/-13	-18/-12/-23	Amphetamine -D6
Amphetamine -D6	PI	2.11	142	93/125/94	-10/-29/-10	-22/-15/-25	-10/-24/-11	-
Cocaine	PI	3.62	304	182/82/105	-14/-27/-20	-13/-32/-36	-21/-28/-14	Cocaine-D3
Cocaine-D3	PI	3.62	307	185/77/91	-14/-23/-10	-20/-55/-48	-20/-26/-19	-
Heroin	PI	3.46	370	165/268/58	-11/-27/-24	-53/-27/-32	-29/-22/-13	Heroin-D3
Heroin-D3	PI	3.46	373	165/271/331	-25/-18/-19	-50/-29/-28	-30/-30/-24	-
GBL	PI	1.42	87	45/43	-16/-15	-15/-28	-18/-17	GHB-D6
GHB	PI	0.75	105	87/45/43	-18/-19/-20	-10/-20/-15	-17/-18/-17	GHB-D6
GHB-D6	PI	0.75	111	93/49	-18/-20	-10/-15	-17/-17	-
ketamine	PI	3.33	238	125/220/179	-30/-25/-28	-29/-15/-18	-23/-16/-21	MDMA-D3
LSD	PI	3.92	324	223/207/208	-23/-23/-23	-23/-44/-29	-25/-23/-23	LSD-D3
LSD-D3	PI	3.92	327	226/210/211	-23/-16/-17	-26/-48/-35	-25/-23/-23	-
MDMA	PI	2.66	194	163/105/135	-24/-23-/-24	-13/-25/-20	-29/-19/-25	MDMA-D3
MDMA-D3	PI	2.66	197	163/105/135	-24/-21/-23	-15/-25/-23	-18/-20/-26	-
Methamphetamine	PI	2.43	150	91/65/119	-29/-10/-29	-22/-39/-15	-30/-12 /-30	Methamphetamine -D5
Methamphetamine -D5	PI	2.43	155	92/91/121	-29/-11/-30	-20/-16/-16	-30/-10/-23	-
Morphine	PI	1.80	286	152/201/128	-14/-10/-17	-55/-27/-54	-28/-21/-21	Morphine -D3
Morphine -D3	PI	1.80	289	152/201/165	-21/-30/-29	-55/-27/-42	-28 / -22 / -30	-
trans-Δ9-THC	PI	4.68	315	193/123/259	-16/-15/-16	-25/-34/-20	-21 / -23 / -28	trans-Δ9-THC -D3
THCA	NI	4.76	357	313/245/191	11/13/14	24/31/33	14 / 11 / 19	trans-Δ9-THC -D3
trans-Δ9-THC -D3	PI	4.68	318	196/123/262	-12/-12/-24	-25/-35/-22	-21 / -24 / -28	-
